# Hollow Mesoporous Silica Nanoparticles Co-Loaded with Docetaxel and Indocyanine Green for Synergistic Chemo–Photothermal Therapy

**DOI:** 10.3390/nano16130805

**Published:** 2026-06-30

**Authors:** Guangru Chu, Kaiyi Zhang, Yaru Wu, Siqi He, Zhongkai Liu, Aijiao Wang, Hongji Wang, Liying Cui, Shengkai Liu, Jin Huang, Jinsong Peng, Zhiguo Liu

**Affiliations:** 1 Key Laboratory of Forest Plant Ecology, Ministry of Education, Northeast Forestry University, Harbin 150040, China; 2 College of Food and Health, Northeast Forestry University, Harbin 150040, China; 3 College of Chemistry, Chemical Engineering and Resource Utilization, Northeast Forestry University, Harbin 150040, China; 4 Engineering Research Center of Forest Bio-Preparation, Ministry of Education, Northeast Forestry University, Harbin 150040, China; 5 Heilongjiang Provincial Key Laboratory of Ecological Utilization of Forestry-Based Active Substances, Northeast Forestry University, Harbin 150040, China

**Keywords:** hollow mesoporous silica nanoparticles, docetaxel, indocyanine green, drug delivery, chemo–photothermal synergistic therapy

## Abstract

Hollow mesoporous silica nanoparticles (HSNs) were synthesized via the Stöber method using resorcinol–formaldehyde resin as a template and further developed as a multifunctional nanocarrier for synergistic chemo–photothermal therapy. Docetaxel (DTX) and indocyanine green (ICG) were co-loaded into HSNs as the prodrug and photothermal agent. The loading sequence of these agents can critically affect encapsulation efficiency. Preloading DTX followed by ICG incorporation achieved the highest drug loading (38.65%) and preserved the photoactivity of ICG. The resulting ICG&DTX@NH_2_-HSNs exhibited strong and stable near-infrared photothermal conversion, as well as pH- and laser-responsive drug release behavior. In vitro studies confirmed efficient cellular uptake by 4T1 tumor cells and enhanced cytotoxicity compared with single treatments. In vivo experiments demonstrated significant tumor growth suppression in 4T1 tumor-bearing mice, with the greatest effect observed under combined ICG&DTX@NH_2_-HSNs and laser irradiation. Importantly, histological analysis of major organs revealed no obvious toxicity, confirming the biosafety of the present nanoplatform. This study confirmed the potential of hollow mesoporous silica-based nanocarriers as safe and effective platforms for combined chemotherapy and photothermal cancer therapy.

## 1. Introduction

Cancer, a disease with an extremely high fatality rate worldwide, has rapidly become one of the leading causes of human death [1]. Among the variety of cancers, breast cancer, with its high incidence and mortality rates, continues to pose a severe challenge to global public health [2]. Currently employed clinical strategies for tumor treatment, including surgery, radiotherapy, chemotherapy, targeted therapy, and immunotherapy, can inhibit tumor progression to some extent. However, these approaches often induce severe side effects during the treatment process, causing additional harm to patients’ health [3,4,5]. Among chemotherapeutic agents, docetaxel (DTX) is widely used in the treatment of breast cancer due to its potent antimitotic activity [6]. However, its clinical application is hindered by poor solubility, rapid clearance, and adverse side effects, necessitating the development of advanced drug delivery systems to improve therapeutic efficacy and safety [7,8].

In recent years, nanotechnology-based drug delivery platforms have attracted considerable attention for their ability to enhance drug solubility, prolong systemic circulation, and achieve targeted release at tumor sites [9,10,11,12]. Among these nanocarriers, mesoporous silica nanoparticles (MSNs) stand out due to their tunable pore size, high surface area, excellent chemical stability, and facile surface modification [13,14,15,16]. The hollow mesoporous silica nanoparticle (HSN) design further provides a large cavity volume for high drug loading, making it a promising vehicle for the co-delivery of multiple therapeutic agents [17,18,19]. Importantly, the biocompatibility and biodegradability of silica materials ensure their safety for biomedical applications [20,21,22].

To achieve enhanced therapeutic outcomes, chemo–photothermal combination therapy has emerged as a powerful strategy [23,24,25]. Photothermal therapy (PTT) has been widely considered as an effective treatment in recent years. The photothermal agent converts irradiated near-infrared light into heat to destroy the structure of cancer cells [26]. However, the photothermal agent is the key factor in PTT, and the selection and use of photothermal agents are crucial to the success of PTT [27,28]. Indocyanine green (ICG), an FDA-approved NIR dye, is widely applied as a photothermal agent due to its strong NIR absorption and fluorescence imaging capability [29]. However, ICG suffers from instability, rapid clearance, and photobleaching, which limit its standalone therapeutic application [30,31]. Incorporating ICG into nanocarriers not only stabilizes its photothermal performance but also allows for its integration with chemotherapy to achieve synergistic effects [32,33,34].

In this study, we developed HSNs for the co-delivery of DTX and ICG. By systematically investigating the loading sequence, we demonstrated that preloading DTX followed by ICG incorporation achieved superior drug encapsulation efficiency and structural stability. The resulting ICG&DTX@NH_2_-HSNs exhibited robust pH- and NIR-responsive drug release, efficient cellular uptake by 4T1 breast cancer cells, and enhanced in vitro cytotoxicity compared to single-modality treatments. Furthermore, in vivo studies on 4T1 tumor-bearing mice revealed potent tumor inhibition with minimal systemic toxicity, confirming the potential of this nanoplatform as a safe and effective strategy for synergistic chemo–photothermal cancer therapy.

## 2. Materials and Methods

### 2.1. Materials

Resorcinol (R, AR), cetyltrimethylammonium bromide (CTAB, AR), tetraethyl orthosilicate (TEOS, AR), indocyanine green (ICG, 95%), and docetaxel (DTX, ≥98%) were purchased from Shanghai Sangon Biotech (Shanghai, China) and used as received without further purification. Ammonia solution, ethanol, anhydrous ethanol, and phosphate-buffered saline (PBS, pH 7.4) were supplied by BioSharp Biotechnology Co., Ltd. (Beijing, China), while dimethyl sulfoxide (DMSO) and 3-Aminopropyltriethoxysilane(APTES) were purchased from Aladdin Reagent Co., Ltd. (Shanghai, China). The MTT Cell Proliferation and Cytotoxicity Assay Kit and Annexin V-FITC/PI Apoptosis Detection Kit were purchased from Beyotime Biotechnology (Shanghai, China). L929 fibroblast cells and 4T1 breast cancer cells were purchased from the Cell Bank of the Chinese Academy of Sciences (Shanghai, China). Female Kunming mice (15–20 g) were obtained from the Second Affiliated Hospital of Harbin Medical University (Harbin, China). Unless otherwise specified, all reagents and solvents were of analytical grade and used directly without further purification.

The microstructure and morphology of the materials were characterized by scanning electron microscopy (SEM; JSM-7800F, JEOL Ltd., Japan) and transmission electron microscopy (TEM; JEM-2100, JEOL Ltd., Japan). DTX was quantified using an ultra-high-performance liquid chromatography system (1290 Infinity, Agilent Technologies, USA). Particle size was measured by dynamic light scattering (DLS) using a laser particle size analyzer (UK). Apoptosis and tumor cell viability were analyzed by flow cytometry using a CytoFLEX flow cytometer (USA). Cell images were acquired using a Cytation 5 high-content cell imaging system (USA).

### 2.2. Synthesis of Amino-Functionalized Hollow Mesoporous Silica Nanoparticles (NH_2_-HSNs)

NH_2_-HSNs were synthesized based on a modified Stöber method using resorcinol–formaldehyde resin as the template, cetyltrimethylammonium bromide (CTAB) as the pore-forming agent, and tetraethyl orthosilicate (TEOS) as the silica source under ammonia catalysis [35,36]. Briefly, 0.3 g of resorcinol and 0.4 mL of formaldehyde were dissolved in an ammonia-water-ethanol reaction system to form resorcinol–formaldehyde resin nanoparticles as soft templates. Subsequently, 0.2 g of CTAB was added as the pore-forming agent, and the mixture was stirred thoroughly. Then, 1.2 mL of TEOS was added, and the reaction was continued for 4–5 h. The precipitate was collected by centrifugation and washed three times with anhydrous ethanol and deionized water. The obtained product was dried and then calcined in a muffle furnace at 550 °C for 6 h to remove the template, yielding white HSN powder.

For amino functionalization, 100 mg of HSNs was dispersed in a mixed solution containing 40 mL of anhydrous ethanol and 10 mL of deionized water under magnetic stirring at room temperature. The pH of the suspension was adjusted to 4–5 with acetic acid, and 1 mL of APTES was slowly added dropwise. The reaction was continued under stirring at room temperature for 5 h. The product was collected by centrifugation at 8000 rpm for 10 min, washed three times with ethanol and deionized water, and finally dried under vacuum to obtain NH_2_-HSNs as a white powder.

### 2.3. Loading of Indocyanine Green (ICG) and Docetaxel (DTX)

For drug loading, 20 mg of NH_2_-HSNs was dispersed in 20 mL of anhydrous ethanol to obtain a 1 mg/mL NH_2_-HSN suspension. DTX was dissolved in dimethyl sulfoxide (DMSO) and first added to the NH_2_-HSN suspension at different DTX mass ratios of 1:1, 2:1, and 1:2. The mixture was stirred at room temperature in the dark for 12 h. The DTX-loaded nanoparticles were then collected by centrifugation and gently washed twice with PBS buffer (pH 7.4) to remove surface-adsorbed drug molecules. The obtained DTX@NH_2_-HSNs were re-dispersed in 5 mL of PBS buffer (pH 7.4), after which ICG was added and the mixture was stirred in the dark at room temperature for another 12 h. The dual drug-loaded nanoparticles, denoted as ICG&DTX@NH_2_-HSNs, were collected by centrifugation, washed twice with PBS, and freeze-dried for further use.

To investigate the influence of the loading sequence on drug loading performance, another loading strategy was also performed, during which ICG was loaded first, followed by DTX loading under the same experimental conditions. After drug loading, the supernatants obtained after centrifugation were collected and analyzed to calculate the drug loading capacity and encapsulation efficiency of DTX and ICG.

$$LE\left(\%\right)=\frac{weight\;of\;loaded\;drug}{weight\;of\;(HSNs+loadeddrug)}\times 100\%$$

$$EE\left(\%\right)=\frac{weight\;of\;loaded\;drug}{weight\;of\;initial\;total\;drug}\times 100\%$$

### 2.4. DTX Is Released from NH_2_-HSNs

For the in vitro release study, only DTX release from ICG&DTX@NH_2_-HSNs was quantitatively monitored, while ICG served as the photothermal agent for NIR-triggered release. Briefly, 2 mg of each formulation was accurately weighed, dispersed in a 4 mL PBS buffer by ultrasonication, and transferred into a dialysis bag with a molecular weight cut-off of 3500 Da. The dialysis bag was then immersed in 10 mL of release medium at different pH values and gently stirred at 37 °C. At predetermined time points (1, 2, 4, 6, 8, 10, 12, 24, 36, and 48 h), 2 mL of release medium was collected and immediately replaced with an equal volume of fresh PBS.

The collected samples were analyzed by HPLC to determine the released DTX concentration. A Diamonsil C18 reversed-phase column (250 mm × 4.6 mm, 5 μm) was used for separation. The mobile phase consisted of methanol, acetonitrile, and water (35:40:25, *v*/*v*/*v*), and separation was performed under isocratic elution. The flow rate was 1.0 mL/min, the detection wavelength was 230 nm, the injection volume was 10 μL, the column temperature was maintained at 25 °C, and the run time was 10 min. For the NIR-triggered release group, 808 nm laser irradiation was applied intermittently at a power density of 2.1 W/cm^2^.

$$Er=\frac{V0\times Ct+V\times \sum Ci}{m}\times 100\%$$

Here, the following variables are defined: *E*r: cumulative release amount of DTX (%); *V*0: total volume of the releasing medium (mL); ∑*C*i: sum of the concentration of the drug in the releasing medium from the first sampling time point to the last sampling moment (μg/mL); *V*: volume of each sampling (mL); m: total mass of the drug in the formulation (mg).

### 2.5. In Vitro Cell Experiment

#### 2.5.1. Cytotoxicity Assay

To assess the cytotoxicity of NH_2_-HSNs, L929 cells were cultured in a 96-well plate with five parallel experiments for each group. Then, 100 μL of cell volume was added to each well, and when the cell confluence reached approximately 80%, HSNs at concentrations of 5, 10, 15, 20, and 25 μg/mL were introduced. The control group received an equal volume of medium with a concentration of 0 μg/mL. The plates were incubated in an incubator (37 °C, 5% CO_2_) for 12 h. After discarding the culture medium, 10 μL of the 0.5 mg/mL MTT solution was added to each well. Following 4 h incubation in the incubator, 100 μL of DMSO was added to each well, and absorbance was measured using a microplate reader.

$$Cell\;viability=\frac{Experimental\;group}{Blank\;group}\times 100\%$$

#### 2.5.2. In Vitro Cell Uptake Experiment

Cultivate L929 and 4T1 cells in a six-well plate for 24 h. Begin cell experiments when cell coverage reaches 85%. Discard the old culture medium and wash three times with an appropriate amount of physiological saline. Add ICG&DTX@ NH_2_-HSNs and continue the culture for 4 h. After washing three times with PBS, fix with 4% paraformaldehyde for 10 min and stain with PI for 15 min. The cellular uptake behavior was observed using a Cytation 5 high-content cell imaging analysis system (BioTek Instruments, USA).

#### 2.5.3. Cell Flow Cytometry

Seed 4T1 cells onto a 6-well culture plate and incubate them in an incubator for 24 h. After the cells have adhered, treat them with DTX, HSNs, ICG&DTX@ NH_2_-HSNs, and ICG&DTX@ NH_2_-HSNs (with 808 nm, 2.1 W/cm^2^ laser irradiation for 5 min), and after 24 h of treatment, wash the cells three times with PBS, digest with trypsin, and stain with Annexin-V-FITC/PI. The stained cells were analyzed using a CytoFLEX flow cytometer (Beckman Coulter, USA).

### 2.6. In Vivo Activity Experiment

Female Kunming mice (15~20 g) were obtained from the Second Affiliated Hospital of Harbin Medical University (Harbin, China). All animal procedures were approved by the Laboratory Animal Management and Ethics Committee of Northeast Forestry University. All experiments were conducted in accordance with the institutional guidelines (Approval No. 2025019) issued by the Laboratory Animal Management and Ethics Committee of Northeast Forestry University. 4T1 tumor cells were sourced from the Cell Bank of the Chinese Academy of Sciences (Shanghai, China). Cells with a tumor density of 80–90% were initially diluted in sterile physiological saline to a concentration of 1 × 10^7^ cells/mL. Subsequently, the tumor cells were injected subcutaneously into the right forearm of each mouse. The ectopic subcutaneous forearm model was selected because it enables reproducible tumor establishment, convenient tumor volume measurement, and localized NIR irradiation for evaluating photothermal treatment efficacy, although orthotopic mammary fat pad models are more representative of the breast tumor microenvironment. Subsequently, the mouse models with breast cancer were randomly divided into five treatment groups (n = 6): The control group received an injection of normal saline (NaCl, 0.2 mL); the free DTX group was administered docetaxel solution at 10 mg/kg (0.2 mL); the HSN group received blank HSNs at 18.0 mg/kg (0.2 mL); the ICG&DTX@NH_2_-HSN group was administered 28.0 mg/kg of the nanocomposite (corresponding to 10 mg/kg DTX, 0.2 mL); the ICG&DTX@NH_2_-HSN + NIR group received the same dose of nanocomposite followed by 808 nm, 2.1 W/cm^2^ laser irradiation. Thereafter, the body weight of each mouse was recorded every two days following the initial administration. Two weeks after treatment, the mice were euthanized, and their tumors and the heart, liver, and kidneys were collected for further analysis.

## 3. Results and Discussion

### 3.1. HSN Synthesis and Characterization

**Figure 1:**
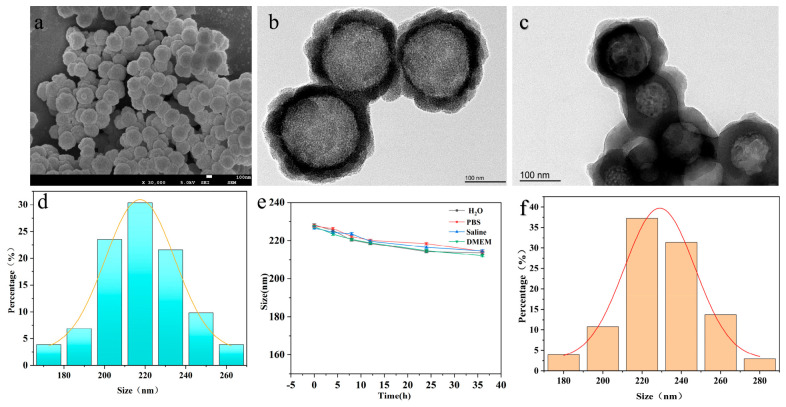
(**a**) SEM image of HSNs; (**b**) TEM image of HSNs; (**c**) TEM image of ICG&DTX@ NH_2_-HSNs; (**d**) DLS results of HSNs; (**e**) variation in particle size of HSNs in different media; (**f**) DLS results of ICG&DTX@ NH_2_-HSNs.

The morphology and structural features of the synthesized HSNs were systematically characterized by SEM, TEM, and DLS. As shown in Figure 1a, the as-synthesized nanoparticles exhibited uniform spherical morphology with a smooth surface. The TEM image in Figure 1b further revealed a well-defined shell with mesoporous channels and a big hollow interior. This hollow mesoporous structure provides both a large internal cavity for drug encapsulation and abundant mesopores for guest molecule adsorption, which are essential characteristics for an efficient nanocarrier system.

The hydrodynamic diameters of HSNs, NH_2_-HSNs, and ICG&DTX@NH_2_-HSNs were determined by DLS after dispersion in ethanol. DLS measurement results are shown in Figure 1d; the average diameter of the obtained HSNs is 220.0 ± 5.0 nm. Figure 1e displays the changes in particle size of HSNs under different storage conditions. After storage in different media for 36 h, the size of the HSNs was slightly decreased, which can be ascribed to the surface of HSNs being enveloped by solvent molecules after dissolving the unstable segment, resulting in a certain degree of shielding of the particle surface. This stable size characteristic is of significant importance for the design and application of nanocarriers, ensuring their persistent stability and controllable release properties under physiological environmental conditions.

Figure 1c shows the representative TEM image of ICG&DTX@ NH_2_-HSNs. There are no significant structural changes after loading of DTX and ICG in comparison to those of HSNs.

The average hydrodynamic diameter of HSNs is shown in Figure 1f; the size was increased from approximately 220.0 ± 5.0 nm to 230.0 ± 5.0 nm after drug loading. This slight size enlargement indicates the successful incorporation of drug molecules into the mesoporous framework and partial adsorption on the nanoparticle surface.

**Figure 2:**
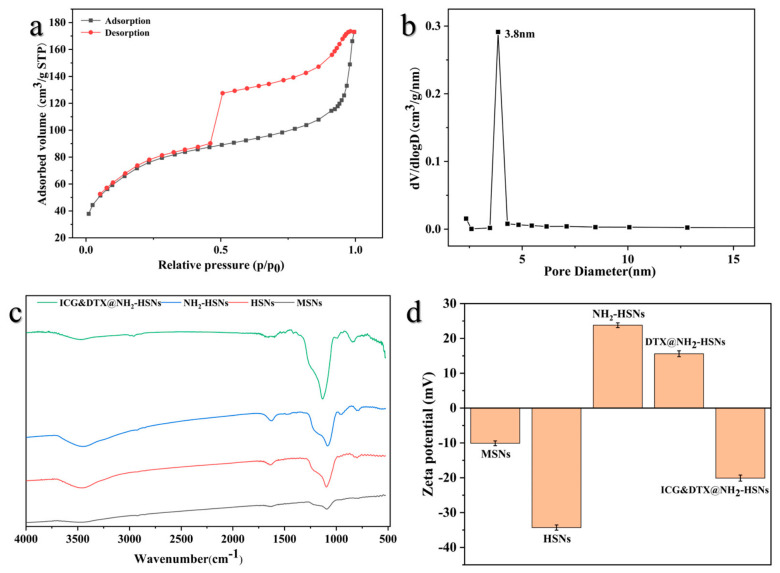
(**a**) Nitrogen adsorption–desorption isotherms of HSNs; (**b**) pore size distribution of HSNs; (**c**) FTIR spectra of MSNs, HSNs, and NH_2_-HSNs; (**d**) the zeta potentials of samples during different stages of synthesis.

Nitrogen adsorption-desorption analysis was performed to evaluate the textural properties of the synthesized HSNs. As presented in Figure 2a, the isotherm displayed a typical type IV adsorption-desorption profile with a distinct hysteresis loop, which is characteristic of mesoporous materials. The pronounced nitrogen uptake at high relative pressure further suggests the presence of hollow cavities and interparticle voids. Moreover, in Figure 2b, the pore size distribution curve revealed a dominant pore diameter of approximately 3.8 nm, indicating a relatively uniform mesoporous structure. The formation of such mesopores can be attributed to the removal of the template and pore-forming agent during calcination. These structural features provide abundant adsorption sites and diffusion channels for drug molecules, which are essential for achieving efficient drug loading and controlled release. Taken together, the nitrogen adsorption–desorption results confirm the successful construction of hollow mesoporous silica nanoparticles with appropriate pore dimensions for drug delivery applications.

FTIR spectroscopy was employed to verify the surface functionalization and drug loading of the synthesized nanoparticles, as shown in Figure 2c. All samples exhibited typical absorption bands of the silica framework at approximately 1080 cm^−1^ and 800 cm^−1^, which were assigned to the asymmetric and symmetric stretching vibrations of Si-O-Si, respectively. The weak band near 960 cm^−1^ was attributed to the stretching vibration of surface Si-OH groups, indicating the presence of hydroxyl groups on the silica surface. Compared with MSNs and HSNs, NH_2_-HSNs showed additional absorption features in the regions of 3300–3500 cm^−1^ and 1560–1650 cm^−1^, corresponding to the N-H stretching and bending vibrations of amino groups, respectively. In addition, the weak absorption band around 2930 cm^−1^ was associated with the -CH_2_- stretching vibration from APTES, further confirming the successful grafting of amino groups onto the HSN surface.

After the sequential loading of DTX and ICG, the FTIR spectrum of ICG&DTX@NH_2_-HSNs retained the characteristic Si-O-Si absorption bands, indicating that the silica framework remained intact during the loading process. Meanwhile, several strengthened absorption bands appeared in the organic vibration regions, particularly around 1600–1500 cm^−1^ and 1300–1000 cm^−1^, which could be attributed to the characteristic vibrations of DTX and ICG molecules, including C=O, C=C/C-N, and related functional groups. These spectral changes demonstrate the successful amino-functionalization of HSNs and the subsequent incorporation of DTX and ICG into the NH_2_-HSN nanocarrier.

The surface charge evolution of the nanoparticles during calcination and sequential drug loading was evaluated by zeta potential measurements. As shown in Figure 2d, the zeta potential of MSNs was about −10.1 mV, which decreased to −35 mV after forming HSNs, suggesting increased exposure of silanol groups after template removal. After amine modification, the surface potential shifted dramatically to +23.8 mV, confirming successful grafting of amino groups on the nanoparticle’s surface. Following DTX loading, the potential slightly decreased to about +15.6 mV, which may be attributed to partial coverage of the surface amino groups by DTX molecules. Finally, after co-loading ICG and DTX, the zeta potential shifted to approximately −20.1 mV, mainly due to the negatively charged -SO_3_^−^ groups of ICG. These results clearly demonstrate the successful stepwise construction of the drug-loaded nanoplatform.

### 3.2. HSN Loading of DTX and In Vitro Release Studies

**Figure 3:**
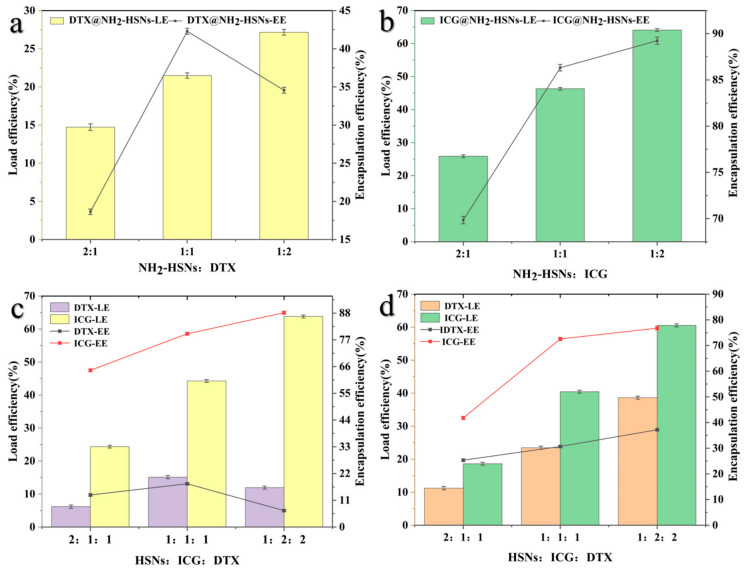
(**a**) DTX@ NH_2_-HSN system; (**b**) ICG@ NH_2_-HSN system; (**c**) ICG loaded first and then the DTX system; (**d**) DTX loaded first and then the ICG system.

A higher drug encapsulation efficiency is a prerequisite for achieving delivery. The simultaneous loading of two drugs may lead to some competition or synergy. Considering that HSNs have a large specific surface area and hollow structure, drugs with different solubilities can be loaded on the shell and hollow core. As shown in Figure 3a, more DTX molecules can load on the NH_2_-HSNs as the proportion of DTX increases, resulting in increased drug loading capacities, with the maximum loading efficiency reaching 27.16 ± 0.38%. However, the encapsulation efficiency does not always increase with an increase in DTX content. The encapsulation efficiency reaches a maximum of 34.56 ± 0.40% at a 1:1 ratio. This means that a further increase in DTX may not be effectively encapsulated inside NH_2_-HSNs. There exists a balance between the loading rate and encapsulation efficiency of DTX by HSNs. As for the loading of ICG into NH_2_-HSNs, as depicted in Figure 3b, the loading efficiency of ICG was increased with changes in the mass ratio of HSNs to ICG from 2:1 to 1:2. The high loading capacity can be attributed to the strong electrostatic interactions between the negatively charged sulfonate groups (-SO_3_^−^) of ICG and the positively charged amino groups on the surface of NH_2_-HSNs, as well as the large internal cavity and mesoporous shell structure of the nanoparticles, which provide sufficient space for dye adsorption.

The loading sequence can significantly influence the loading rate and encapsulation rate of DTX and ICG. With respect to the results of the loading of ICG and then DTX, as shown in Figure 3c, it can be seen that the maximum loading efficiency of DTX reaches 15.11% ± 0.41%. This can be attributed to the preferential loading of ICG molecules forming a physical barrier at the entrance of the channels, which partially obstructs the pathway for DTX to diffuse from the bulk solution into the hollow cavity. As a result, most DTXs can only be loaded onto the exterior shell of the mesoporous channels. The result of the loading of DTX before ICG, shown in Figure 3d, indicated that the maximum loading rate of DTX can reach 38.65 ± 0.45%. This result can be explained as follows. DTX is efficiently loaded into the interior cavity of HSNs through hydrophobic interactions, followed by physical adsorption to stabilize ICG in the outer area of the carrier, thereby maximizing and differentiating the utilization of the carrier space. This strategy not only significantly increases the drug loading capacity of DTX but also ensures the efficient loading of ICG, laying a solid material foundation for constructing an efficient chemotherapy–photothermal synergistic therapy nanoplatform. This result fully demonstrates the decisive role of the loading sequence in the design of multi-drug delivery systems.

**Figure 4:**
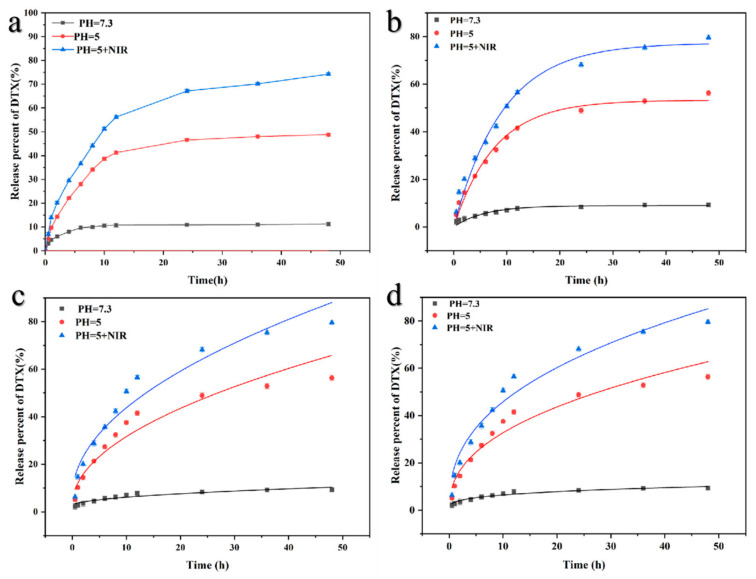
(**a**) Release curves of DTX under laser and non-laser irradiation for ICG&DTX@ NH_2_-HSNs at pH 5.0 and pH 7.4; (**b**) fitting results under the first-order model; (**c**) fitting results under the Higuchi model; (**d**) fitting results under the Ritger–Peppas model.

In vitro release studies revealed strong pH-responsive and photo-responsive behavior, as shown in Figure 4a. At an acidic pH of 5.0, which mimics the tumor microenvironment, DTX exhibited substantially accelerated release in comparison to that of a physiological pH of 7.4. Upon 808 nm NIR irradiation, the release rate was further boosted, reaching 74.23% within 48 h. This enhancement was attributed to the photothermal effect of ICG, which induced localized heating, promoted the disruption of the silica matrix, and facilitated drug diffusion.

The in vitro DTX release data were fitted with first-order kinetics, the Higuchi model, and the Ritger–Peppas kinetic model. Figure 4b–d present the linear fitting curves, which provide the kinetic model equations for the DTX release profiles. The first-order kinetics model (R^2^ > 0.95), Higuchi model (R^2^ > 0.90), and Ritger–Peppas model (R^2^ > 0.92) all demonstrated excellent linear correlations. The release exponent (n) for DTX was less than 0.45 in all cases, indicating that the drug release mechanism from ICG&DTX@ NH_2_-HSNs is Fickian diffusion. This suggests that the drug release rate is governed solely by the drug’s diffusion concentration gradient.

### 3.3. The Photothermal Performance of ICG&DTX@ NH_2_-HSNs

**Figure 5:**
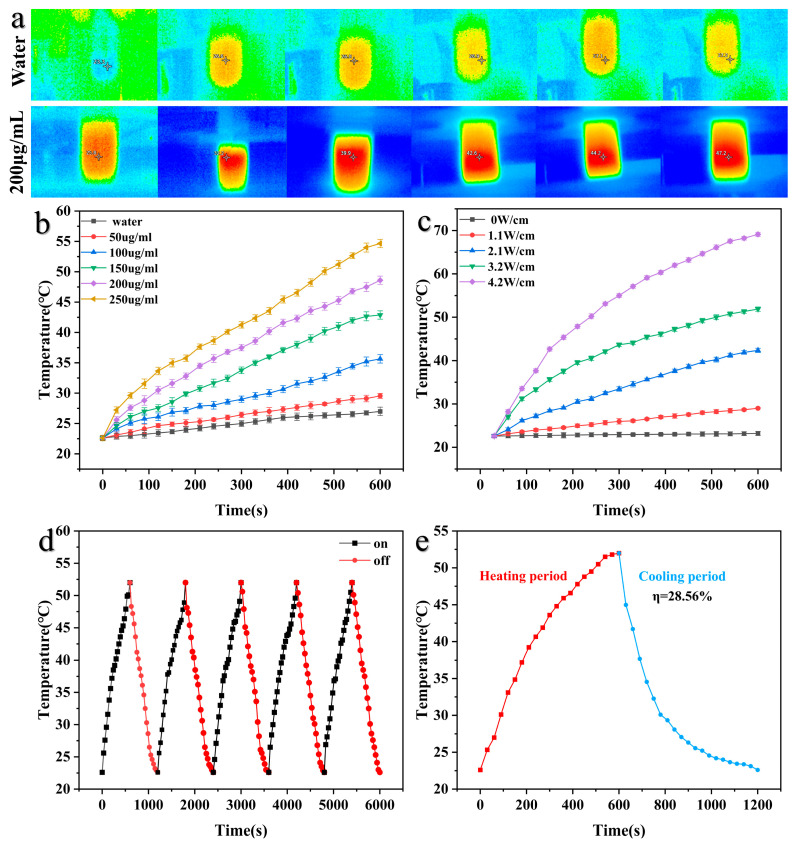
(**a**) Thermal images of ICG&DTX@ NH_2_-HSNs under 808 nm irradiation (2.1 W/cm^2^, 10 min). (**b**) Heating curves of different concentrations of ICG&DTX@ NH_2_-HSNs. (**c**) Heating curves of ICG&DTX@ NH_2_-HSNs (200 μg/mL) under various laser powers. (**d**) Photothermal stability of ICG&DTX@ NH_2_-HSNs over five irradiation cycles. (808 nm, 2.1 W/cm^2^). (**e**) The heating/cooling experiment of ICG&DTX@NH_2_-HSNs under laser (808 nm, 2.1 W/cm^2^) irradiation.

The photothermal properties of ICG&DTX@ NH_2_-HSNs were systematically evaluated under 808 nm near-infrared (NIR) laser irradiation. As shown in Figure 5a, the thermal imaging results clearly demonstrated a rapid temperature rise in ICG&DTX@NH_2_-HSN suspensions upon NIR irradiation, confirming their excellent light-to-heat conversion capability. A concentration-dependent heating effect was observed (Figure 5b), where higher nanoparticle concentrations resulted in more pronounced temperature elevations. This finding highlights the strong photothermal response of ICG encapsulated within the mesoporous silica matrix, ensuring sufficient thermal output for tumor ablation [24].

Furthermore, the heating behavior was positively correlated with laser power density (Figure 5c). The ICG&DTX@NH_2_-HSN solution temperature increased sharply with an increase in irradiation intensity, indicating that ICG&DTX@NH_2_-HSNs can achieve tunable photothermal effects by adjusting external laser parameters. Importantly, repeated irradiation cycles revealed minimal loss of heating capacity (Figure 5d), demonstrating the excellent photothermal stability and reusability of the nanoplatform. This photothermal durability is crucial for clinical translation, where multiple rounds of laser exposure may be required for effective tumor eradication.

To further confirm thermal responsiveness, heating-cooling cycles were performed (Figure 5d). The reversible temperature changes across multiple on/off cycles indicate that the photothermal effect is not due to structural degradation of the nanocarrier but rather originates from the stable incorporation of ICG. Taken together, these findings demonstrate that the synergistic design of high-efficiency DTX encapsulation combined with stable ICG-mediated photothermal conversion provides a powerful strategy for chemo–photothermal therapy. The light-triggered enhancement of DTX release under acidic conditions further highlights the potential of ICG&DTX@ NH_2_-HSNs to achieve on-demand, localized, and tumor-selective drug delivery while minimizing systemic side effects.

### 3.4. In Vitro Cytotoxicity of HSNs and ICG&DTX@ NH_2_-HSN Nanocomposites

**Figure 6:**
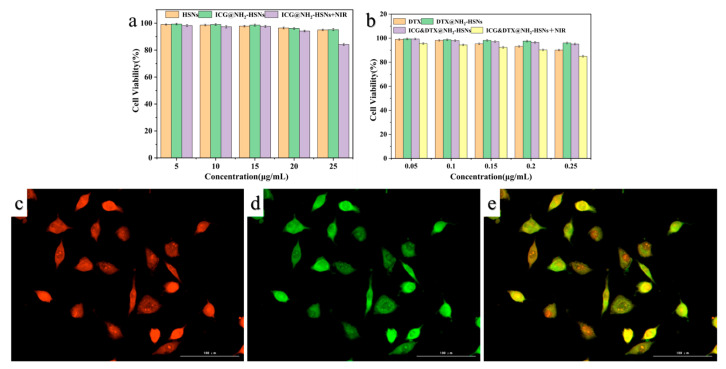
(**a**) Toxicity tests on L929 cells; (**b**) toxicity tests on L929 cells; (**c**) PI staining of L929 cells; (**d**) ICG staining of L929 cells; (**e**) merged images.

The biocompatibility of the HSNs was first assessed using L929 fibroblast cells. As shown in Figure 6c, propidium iodide (PI) staining revealed minimal red fluorescence signals, suggesting negligible membrane damage or necrosis in cells treated with HSNs. The uptake signal of ICG showed green fluorescence (Figure 6d) evenly distributed throughout the cells, with moderate overall intensity and no abnormal aggregation, indicating that the nanoparticles were taken up by the cells. The merged images (Figure 6e) further confirmed that the majority of cells maintained intact morphology and membrane integrity, validating the good cytocompatibility of HSNs.

To quantitatively evaluate the cytotoxicity of the nanoplatform toward normal cells, an MTT assay was performed on L929 fibroblasts. As shown in Figure 5a, cells treated with the blank carrier HSNs at concentrations ranging from 5 to 25 μg/mL maintained high viability above 95%, indicating excellent cytocompatibility. Figure 5b shows the cytotoxicity of L929 cells treated with the drug-loaded nanocomposite ICG&DTX@NH_2_-HSN and ICG&DTX@NH_2_-HSN + NIR under the same concentration range. Cell viability remained above 85% even at the highest concentration (0.25 μg/mL) and after NIR irradiation, demonstrating that the drug-loaded formulation exerts minimal toxicity toward normal fibroblasts. These results indicate that both the HSN carrier and the drug-loaded nanoplatform are biocompatible with normal cells, supporting their use as a safe nanocarrier for combined chemo-photothermal therapy.

**Figure 7:**
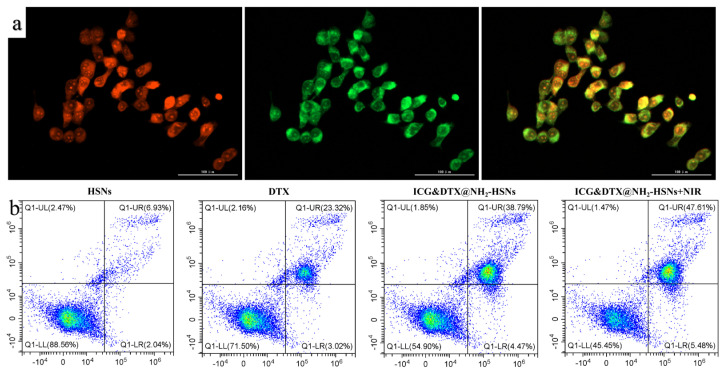
(**a**) Fluorescent images of the uptake of ICG&DTX@ NH_2_-HSNs by 4T1 tumor cells. High-content fluorescent imaging results. (**b**) Flow cytometric analysis of 4T1 cells treated with 25 µg/mL HSNs; 0.20 µg/mL DTX, ICG&DTX@ NH_2_-HSNs, and ICG&DTX@ NH_2_-HSN + NIR.

The cellular internalization of ICG&DTX@ NH_2_-HSNs was investigated with 4T1 breast tumor cells using a high-content fluorescence imaging system. As shown in Figure 7a, a strong intracellular fluorescence signal was observed after 4 h of incubation, indicating that the nanocarriers were efficiently taken up by tumor cells. The fluorescence distribution was primarily localized in the cytoplasmic region, suggesting that ICG&DTX@ NH_2_-HSNs were internalized through endocytosis rather than passive membrane diffusion. The clear and widespread fluorescence signals further confirmed that the hollow mesoporous structure facilitated efficient cellular penetration and stable retention, which are essential prerequisites for effective drug delivery and photothermal therapy [37,38].

To further evaluate the therapeutic efficacy of different treatments, Annexin V-FITC/PI flow cytometry analysis was performed on 4T1 cells (Figure 7b). The cell populations were classified as viable cells, early apoptotic cells (Annexin V^+^/PI^−^), late apoptotic cells (Annexin V^+^/PI^+^), and necrotic cells (Annexin V^−^/PI^+^). Free DTX induced a moderate apoptotic response in 4T1 cells, with a total apoptotic rate of 26.34%, including 3.02% early apoptotic cells and 23.32% late apoptotic cells. In contrast, blank HSNs caused only limited apoptosis or necrosis, further confirming the good cytocompatibility of the nanocarrier itself.

Compared with free DTX, ICG&DTX@NH_2_-HSNs induced a higher proportion of apoptotic cells, with 4.47% early apoptosis, 38.79% late apoptosis, and 43.26% necrosis. After 808 nm NIR irradiation, the apoptosis-inducing effect was further enhanced, showing 5.46% early apoptotic cells, 47.61% late apoptotic cells, and 53.07% necrotic cells. The quantitative results are summarized in Figure 6c and statistically analyzed by one-way ANOVA. These results indicate that ICG&DTX@NH_2_-HSNs combined with NIR irradiation enhanced apoptosis induction in 4T1 tumor cells, which may be attributed to the combined effects of localized hyperthermia-enhanced drug release and the intrinsic cytotoxicity of DTX. Overall, these findings demonstrate that ICG&DTX@NH_2_-HSNs can be effectively internalized by tumor cells and exert an enhanced chemo-photothermal therapeutic effect.

### 3.5. In Vivo Antitumor Activity Study

**Figure 8:**
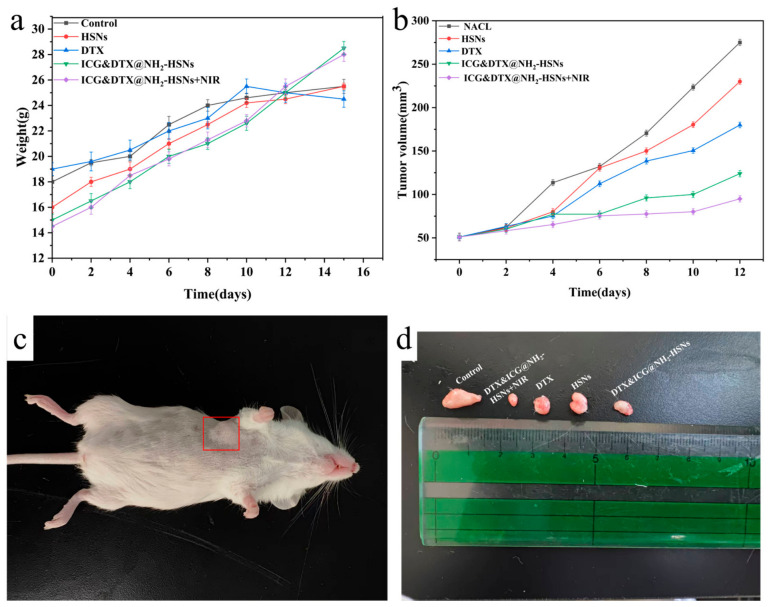
(**a**) Weight change curve of mice after different treatments. (**b**) Results of tumor volume changes after different treatments. (**c**) Schematic illustration of successful mouse modeling(The red box indicates successful 4T1 tumor inoculation in mice). (**d**) Representative images of tumor resection after different treatments.

The therapeutic performance of ICG&DTX@ NH_2_-HSNs was further evaluated on 4T1 tumor-bearing mice. As shown in Figure 8a, no significant body weight loss was observed across all groups during the treatment period, indicating that the nanoplatform exhibited negligible systemic toxicity and good biocompatibility. This is consistent with the in vitro cytotoxicity results, further confirming the biosafety of HSNs as a drug delivery vehicle. The successful establishment of the 4T1 tumor model was confirmed by the representative images shown in Figure 8b, which provided a reliable platform for evaluating the in vivo efficacy of the nanoplatform. Tumor growth inhibition was systematically analyzed (Figure 8c). The control group displayed rapid tumor progression, while free DTX and blank HSNs produced only limited suppression of tumor growth. In contrast, mice treated with ICG&DTX@ NH_2_-HSNs showed markedly reduced tumor volumes, demonstrating the synergistic efficacy of combined chemotherapy and photothermal therapy. Notably, upon exposure to 808 nm laser irradiation, the ICG&DTX@ NH_2_-HSN group exhibited the most pronounced tumor regression, with tumor volumes significantly smaller than those in other groups, highlighting the therapeutic advantage of light-triggered drug release and localized hyperthermia. To further assess the therapeutic efficacy of ICG&DTX@ NH_2_-HSNs, Figure 8d shows the representative images of the excised tumors and major organs collected after two weeks of treatment. As shown in Figure 8d, the control group exhibited large and intact tumors, consistent with the rapid tumor progression observed in the volume measurements. Free DTX and blank HSN group resulted in only partial suppression of tumor growth, whereas ICG&DTX@ NH_2_-HSNs significantly reduced tumor size. Notably, the most pronounced tumor regression was observed in the ICG&DTX@NH_2_-HSN plus laser group, further confirming the synergistic chemo–photothermal therapeutic effect. Taken together, these results indicate that ICG&DTX@NH_2_-HSNs combined with NIR irradiation achieved an enhanced chemo-photothermal therapeutic effect compared with chemotherapy alone. The ability to achieve significant tumor regression with minimal systemic side effects underscores the promise of this nanoplatform as an effective strategy for synergistic chemo–photothermal cancer therapy.

**Figure 9:**
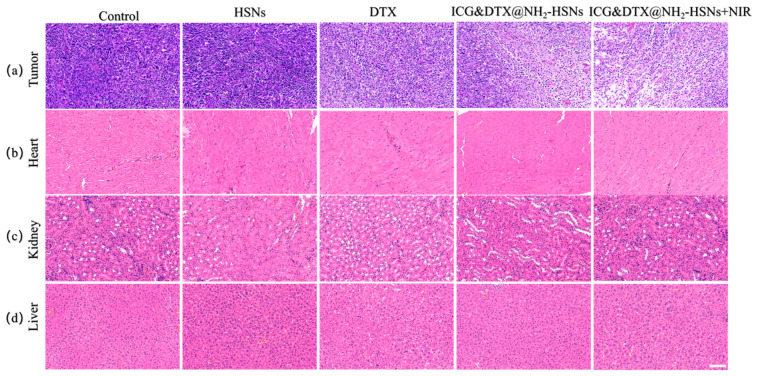
The images of tumors and major organs from each treatment group: (**a**) tumor, (**b**) heart, (**c**) kidney, and (**d**) liver (scale bar: 100 μm).

In addition to tumor inhibition, systemic toxicity was carefully evaluated by examining the histological features of major organs, including the heart, kidney, and liver (Figure 9b–d). No obvious pathological abnormalities, necrosis, or inflammatory infiltrations were detected across all treatment groups. The cardiac tissues (Figure 8b) maintained normal morphology, indicating that nanoformulation and laser irradiation did not induce cardiotoxicity. Similarly, the kidneys (Figure 9c) showed intact glomerular and tubular structures without signs of damage, suggesting that renal function was preserved. The liver sections (Figure 9d) displayed normal hepatic architecture without evidence of hepatocellular injury or fibrosis, confirming the absence of liver toxicity. These results collectively demonstrate that ICG&DTX@NH_2_-HSNs exhibit potent antitumor activity while maintaining excellent biosafety in vivo. The absence of significant histological abnormalities in major organs highlights the biocompatibility of the hollow mesoporous silica nanocarrier and suggests that the synergistic chemo–photothermal strategy could provide effective tumor suppression with minimal systemic side effects.

In comparison with previously reported mesoporous silica-based nanoplatforms [39], which mainly rely on surface adsorption or mesoporous channels for drug loading, the hollow mesoporous structure developed here provides a larger internal cavity and more efficient space utilization, resulting in a markedly higher docetaxel loading efficiency of 38.65 ± 0.45%. In many existing chemo–photothermal systems, co-loading of chemotherapeutic drugs and photothermal agents is achieved without regulating the loading sequence, which often limits synergistic efficacy due to competitive adsorption [40]. The sequence-optimized strategy employed in this study significantly enhances both drug encapsulation and therapeutic performance. Although pH-responsive drug release has been widely reported, cumulative release under acidic conditions typically remains below 60%, whereas the ICG&DTX@NH_2_-HSN system achieves 74.23% release at pH 5.0 under 808 nm irradiation, indicating stronger coupling between photothermal activation and drug diffusion. Moreover, many reported systems exhibit only moderate in vitro cytotoxicity and limited in vivo validation [41]. The present nanoplatform induces 53.09% cell mortality compared with 26.34% for free docetaxel, and it achieves effective tumor suppression in vivo with minimal systemic toxicity.

To further evaluate the short-term hematological safety of the nanoplatform, routine blood analysis was performed after treatment, as shown in Tables S2–S6. The red blood cell-related parameters, including RBC, HGB, and HCT, remained within the normal reference ranges in all groups, indicating that no obvious anemia-related toxicity was observed under the tested conditions. Platelet counts in the ICG&DTX@NH_2_-HSN and ICG&DTX@NH_2_-HSN + NIR groups also remained within the reference range. However, decreases in white-blood-cell-related parameters, including WBC, neutrophils, and lymphocytes, were observed in the DTX-containing treatment groups, suggesting a possible hematological response associated with DTX-containing formulations. Therefore, together with the stable body weight and H&E staining results, these data suggest that ICG&DTX@NH_2_-HSNs did not induce obvious erythrocyte- or platelet-related toxicity during the short-term treatment period. Nevertheless, further serum biochemical analysis, biodistribution, and long-term clearance studies are still required to comprehensively evaluate the systemic biosafety of this nanoplatform.

## 4. Conclusions

In this study, HSNs were successfully synthesized as a multifunctional nanoplatform for the co-delivery of DTX and ICG. After optimizing the loading sequence, preloading DTX followed by ICG incorporation achieved the highest encapsulation efficiency and structural stability. The resulting ICG&DTX@ NH_2_-HSNs displayed pH- and NIR-responsive drug release, efficient photothermal conversion, and excellent stability under repeated irradiation. In vitro studies demonstrated effective cellular uptake by 4T1 tumor cells and enhanced cytotoxicity compared with single treatments. In vivo experiments further confirmed significant tumor growth inhibition with minimal systemic toxicity, especially in the ICG&DTX@ NH_2_-HSN plus laser group. Overall, these findings highlight the potential of HSN-based nanocarriers as safe and effective platforms for synergistic chemo–photothermal cancer therapy.

## Data Availability

The original contributions presented in this study are included in the article. Further inquiries can be directed to the corresponding author.

## Supplementary Materials

The following supporting information can be downloaded at https://www.mdpi.com/article/10.3390/nano16130805/s1. Table S1. DTX and ICG loading capacity and encapsulation efficiency under different loading sequences. Table S2. Blood routine test results of mice in the NaCl control group. Table S3. Blood routine test results of mice treated with HSNs alone. Table S4. Blood routine test results of mice treated with DTX alone. Table S5. Blood routine test results of mice treated with ICG&DTX@NH_2_-HSNs. Table S6. Blood routine test results of mice treated with ICG&DTX@NH2-HSNs + NIR.
