# Supplementary material for: Hollow Mesoporous Silica Nanoparticles Co-Loaded with Docetaxel and Indocyanine Green for Synergistic Chemo–Photothermal Therapy
Source: Nanomaterials (Basel). 2026 Jun 30;16(13):805. doi: 10.3390/nano16130805 (PMC13363673; doi:10.3390/nano16130805)
Supplement: Supplementary file 1 [file nanomaterials-16-00805-s001.zip › nanomaterials-4337832-supplementary.pdf]

Table S1 DTX and ICG loading capacity and encapsulation efficiency under different loading sequences.

different drug-loading systems	Ratio	DTX loading efficiency	DTX encapsulation efficiency	ICG loading efficiency	ICG encapsulation efficiency
NH_2-HSNs:DTX	2:1	14.73	18.64		
	1:1	21.49	42.3		
	1:2	27.16	34.56		
NH_2-HSNs:ICG	2:1			25.88	69.84
	1:1			46.32	86.32
	1:2			64.09	89.24
ICG-first loading strategy (NH_2-HSNs:ICG:DTX)	2:1:1	6.19	13.2	24.36	64.44
	1:1:1	15.11	17.8	44.28	79.48
	1:2:2	11.94	6.78	63.81	88.15
DTX-first loading strategy (NH_2-HSNs:ICG:DTX)	2:1:1	11.24	25.33	18.63	41.8
	1:1:1	23.47	30.66	40.46	72.56
	1:2:2	38.65	37.2	60.55	76.75

Table S2 Complete blood count of mice in the NaCl control group

Parameter	Abbr.	Result	Unit	Reference range
White blood cell count	WBC	1.68	10^9/L	0.80 - 10.60
Neutrophil count	Neu	0.36	10^9/L	0.23 - 3.60
Lymphocyte count	Lym	0.85	10^9/L	0.60 - 8.90
Monocyte count	Mon	0.26	10^9/L	0.04 - 1.40
Eosinophil count	Eos	0.20	10^9/L	0.00 - 0.51
Basophil count	Bas	0.01	10^9/L	0.00 - 0.12
Neutrophil percentage	Neu%	20.9	%	6.5 - 50.0
Lymphocyte percentage	Lym%	50.2	%	40.0 - 92.0
Monocyte percentage	Mon%	15.8	%	0.9 - 18.0
Eosinophil percentage	Eos%	12.0	%	0.0 - 7.5
Basophil percentage	Bas%	1.1	%	0.0 - 1.5
Red blood cell count	RBC	8.84	10^{12}/L	6.50 - 11.50
Hemoglobin	HGB	142	g/L	110 - 165
Hematocrit	HCT	46.9	%	35.0 - 55.0
Mean corpuscular volume	MCV	53.1	fL	41.0 - 55.0
Mean corpuscular hemoglobin	MCH	16.0	pg	13.0 - 18.0
Mean corpuscular hemoglobin concentration	MCHC	302	g/L	300 - 360
Red cell distribution width coefficient of variation	RDW-CV	16.9	%	12.0 - 19.0
Red cell distribution width standard deviation	RDW-SD	37.6	fL	23.0 - 39.0

Platelet count	PLT	1289	10^9/L	400 - 1600
Mean platelet volume	MPV	6.6	fL	4.0 - 6.2
Platelet distribution width	PDW	16.0		12.0 - 17.5
Plateletcrit	PCT	0.850	%	0.100 - 0.780

Table S3 Complete blood count of mice in the HSNs-only group

Parameter	Abbr.	Result	Unit	Reference range
White blood cell count	WBC	5.57	10^9/L	0.80 - 10.60
Neutrophil count	Neu	1.81	10^9/L	0.23 - 3.60
Lymphocyte count	Lym	1.10	10^9/L	0.60 - 8.90
Monocyte count	Mon	1.32	10^9/L	0.04 - 1.40
Eosinophil count	Eos	1.13	10^9/L	0.00 - 0.51
Basophil count	Bas	0.21	10^9/L	0.00 - 0.12
Neutrophil percentage	Neu%	32.5	%	6.5 - 50.0
Lymphocyte percentage	Lym%	19.7	%	40.0 - 92.0
Monocyte percentage	Mon%	23.6	%	0.9 - 18.0
Eosinophil percentage	Eos%	20.3	%	0.0 - 7.5
Basophil percentage	Bas%	3.9	%	0.0 - 1.5
Red blood cell count	RBC	8.12	10^{12}/L	6.50 - 11.50
Hemoglobin	HGB	150	g/L	110 - 165
Hematocrit	HCT	46.1	%	35.0 - 55.0
Mean corpuscular volume	MCV	56.8	fL	41.0 - 55.0
Mean corpuscular hemoglobin	MCH	18.5	pg	13.0 - 18.0
Mean corpuscular hemoglobin concentration	MCHC	326	g/L	300 - 360
Red cell distribution width coefficient of variation	RDW-CV	15.5	%	12.0 - 19.0
Red cell distribution width standard deviation	RDW-SD	35.7	fL	23.0 - 39.0
Platelet count	PLT	840	10^9/L	400 - 1600
Mean platelet volume	MPV	6.8	fL	4.0 - 6.2
Platelet distribution width	PDW	16.2		12.0 - 17.5
Plateletcrit	PCT	0.574	%	0.100 - 0.780

Table S4 Complete blood count of mice in the DTX-only group

Parameter	Abbr.	Result	Unit	Reference range
White blood cell count	WBC	1.67	10^9/L	0.80 - 10.60
Neutrophil count	Neu	0.43	10^9/L	0.23 - 3.60
Lymphocyte count	Lym	0.37	10^9/L	0.60 - 8.90
Monocyte count	Mon	0.38	10^9/L	0.04 - 1.40
Eosinophil count	Eos	0.42	10^9/L	0.00 - 0.51

Basophil count	Bas	0.07	10^9/L	0.00 - 0.12
Neutrophil percentage	Neu%	25.7	%	6.5 - 50.0
Lymphocyte percentage	Lym%	22.3	%	40.0 - 92.0
Monocyte percentage	Mon%	22.8	%	0.9 - 18.0
Eosinophil percentage	Eos%	25.0	%	0.0 - 7.5
Basophil percentage	Bas%	4.2	%	0.0 - 1.5
Red blood cell count	RBC	9.19	10^{12}/L	6.50 - 11.50
Hemoglobin	HGB	151	g/L	110 - 165
Hematocrit	HCT	48.5	%	35.0 - 55.0
Mean corpuscular volume	MCV	52.7	fL	41.0 - 55.0
Mean corpuscular hemoglobin	MCH	16.5	pg	13.0 - 18.0
Mean corpuscular hemoglobin concentration	MCHC	312	g/L	300 - 360
Red cell distribution width coefficient of variation	RDW-CV	18.1	%	12.0 - 19.0
Red cell distribution width standard deviation	RDW-SD	39.3	fL	23.0 - 39.0
Platelet count	PLT	358	10^9/L	400 - 1600
Mean platelet volume	MPV	6.1	fL	4.0 - 6.2
Platelet distribution width	PDW	17.0		12.0 - 17.5
Plateletcrit	PCT	0.218	%	0.100 - 0.780

Table S5 Complete blood count of mice in the ICG&DTX@NH_2-HSNs group

Parameter	Abbr.	Result	Unit	Reference range
White blood cell count	WBC	0.47	10^9/L	0.80 - 10.60
Neutrophil count	Neu	0.11	10^9/L	0.23 - 3.60
Lymphocyte count	Lym	0.19	10^9/L	0.60 - 8.90
Monocyte count	Mon	0.15	10^9/L	0.04 - 1.40
Eosinophil count	Eos	0.02	10^9/L	0.00 - 0.51
Basophil count	Bas	0.00	10^9/L	0.00 - 0.12
Neutrophil percentage	Neu%	22.1	%	6.5 - 50.0
Lymphocyte percentage	Lym%	39.8	%	40.0 - 92.0
Monocyte percentage	Mon%	30.9	%	0.9 - 18.0
Eosinophil percentage	Eos%	6.1	%	0.0 - 7.5
Basophil percentage	Bas%	1.1	%	0.0 - 1.5
Red blood cell count	RBC	8.16	10^{12}/L	6.50 - 11.50
Hemoglobin	HGB	137	g/L	110 - 165
Hematocrit	HCT	44.0	%	35.0 - 55.0
Mean corpuscular volume	MCV	54.0	fL	41.0 - 55.0
Mean corpuscular hemoglobin	MCH	16.7	pg	13.0 - 18.0
Mean corpuscular hemoglobin concentration	MCHC	310	g/L	300 - 360
Red cell distribution width	RDW-CV	17.4	%	12.0 - 19.0

coefficient of variation				
Red cell distribution width standard deviation	RDW-SD	39.1	fL	23.0 - 39.0
Platelet count	PLT	1003	10^9/L	400 - 1600
Mean platelet volume	MPV	6.4	fL	4.0 - 6.2
Platelet distribution width	PDW	15.8		12.0 - 17.5
Plateletcrit	PCT	0.644	%	0.100 - 0.780

Table S6 Complete blood count of mice in the ICG&DTX@NH_2-HSNs+NIR group

Parameter	Abbr.	Result	Unit	Reference range
White blood cell count	WBC	0.52	10^9/L	0.80 - 10.60
Neutrophil count	Neu	0.20	10^9/L	0.23 - 3.60
Lymphocyte count	Lym	0.16	10^9/L	0.60 - 8.90
Monocyte count	Mon	0.12	10^9/L	0.04 - 1.40
Eosinophil count	Eos	0.04	10^9/L	0.00 - 0.51
Basophil count	Bas	0.00	10^9/L	0.00 - 0.12
Neutrophil percentage	Neu%	37.6	%	6.5 - 50.0
Lymphocyte percentage	Lym%	29.7	%	40.0 - 92.0
Monocyte percentage	Mon%	22.4	%	0.9 - 18.0
Eosinophil percentage	Eos%	7.3	%	0.0 - 7.5
Basophil percentage	Bas%	1.0	%	0.0 - 1.5
Red blood cell count	RBC	8.42	10^{12}/L	6.50 - 11.50
Hemoglobin	HGB	136	g/L	110 - 165
Hematocrit	HCT	43.4	%	35.0 - 55.0
Mean corpuscular volume	MCV	51.5	fL	41.0 - 55.0
Mean corpuscular hemoglobin	MCH	16.1	pg	13.0 - 18.0
Mean corpuscular hemoglobin concentration	MCHC	313	g/L	300 - 360
Red cell distribution width coefficient of variation	RDW-CV	18.4	%	12.0 - 19.0
Red cell distribution width standard deviation	RDW-SD	39.6	fL	23.0 - 39.0
Platelet count	PLT	1204	10^9/L	400 - 1600
Mean platelet volume	MPV	5.6	fL	4.0 - 6.2
Platelet distribution width	PDW	15.2		12.0 - 17.5
Plateletcrit	PCT	0.679	%	0.100 - 0.780
